# The alluaudite-like arsenate NaCaMg_3_(AsO_4_)_3_

**DOI:** 10.1107/S1600536808014633

**Published:** 2008-05-21

**Authors:** Anissa Haj Abdallah, Amor Haddad

**Affiliations:** a Laboratoire de Matériaux et Cristallochime, Institut Supérieur des Sciences Appliquées et Technologie de Mahdia, Avenue El Mourouj, Sidi Messoud 5111 Hiboun, Mahdia, Tunisia

## Abstract

The title compound, sodium calcium trimagnesium tris­(arsenate), an alluaudite-like arsenate, was prepared by solid-state reaction at high temperature. The structure is built up from edge-sharing MgO_6_ octa­hedra in chains associated with the AsO_4_ arsenate groups. The three-dimensional network leads to two different tunnels occupied statistically by Na^+^ and Ca^2+^. One As and one Mg atom lie on twofold rotation axes; one Na and one Ca are disordered over two sites with occupancies of 0.7 and 0.3 and these sites lie on a twofold rotation axis and an inversion centre, respectively.

## Related literature

For the alluaudite structure type, see: Moore (1971 ▶); Yakubovitch *et al.* (1977 ▶); Cu_1.35_Fe(PO_4_)_3_ (Warner *et al.*, 1993 ▶); NaFe_3.67_(PO_4_)_3_ (Korzenski *et al.*, 1998 ▶). For related alluaudite-like arsenates, see: NaCo_3_(AsO_4_)(HAsO_4_)_2_ (Kwang-Hwa & Pei-Fen, 1994 ▶); NaCaCdMg_2_(AsO_4_)_3_ (Khorari *et al.*, 1997 ▶); Ag_1.49_Mn_1.49_Mn_2_(AsO_4_)_3_ (Ayed *et al.*, 2002 ▶); Na_1.72_ Mn_3.28_(AsO_4_)_3_ (Brahim *et al.* 2003 ▶). For related literature, see: Leroux *et al.* (1995 ▶).

## Experimental

### 

#### Crystal data

NaCaMg_3_(AsO_4_)_3_
*M* *_r_* = 552.74
Monoclinic, 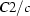

*a* = 11.880 (1) Å
*b* = 12.817 (1) Å
*c* = 6.741 (2) Å
β = 112.45 (1)°
*V* = 948.7 (3) Å^3^
*Z* = 4
Mo *K*α radiation
μ = 11.36 mm^−1^
*T* = 293 (2) K
0.6 × 0.2 × 0.15 mm

#### Data collection

Enraf–Nonius CAD-4 diffractometer
Absorption correction: ψ scan (North *et al.*, 1968 ▶) *T*_min_ = 0.110, *T*_max_ = 0.180
1358 measured reflections
1154 independent reflections
1133 reflections with *I* > 2σ(*I*)
*R*_int_ = 0.021
2 standard reflections frequency: 120 min intensity decay: 0.4%

#### Refinement

*R*[*F* ^2^ > 2σ(*F* ^2^)] = 0.022
*wR*(*F* ^2^) = 0.060
*S* = 1.29
1154 reflections
100 parameters
Δρ_max_ = 0.99 e Å^−3^
Δρ_min_ = −1.00 e Å^−3^

### 

Data collection: *CAD-4 EXPRESS* (Duisenberg, 1992 ▶; Macíček & Yordanov, 1992 ▶); cell refinement: *CAD-4 EXPRESS*; data reduction: *MolEN* (Fair, 1990 ▶); program(s) used to solve structure: *SHELXS86* (Sheldrick, 2008 ▶); program(s) used to refine structure: *SHELXL97* (Sheldrick, 2008 ▶); molecular graphics: *DIAMOND* (Brandenburg, 1998 ▶); software used to prepare material for publication: *SHELXL97* (Sheldrick, 2008 ▶).

## Supplementary Material

Crystal structure: contains datablocks I, global. DOI: 10.1107/S1600536808014633/br2069sup1.cif

Structure factors: contains datablocks I. DOI: 10.1107/S1600536808014633/br2069Isup2.hkl

Additional supplementary materials:  crystallographic information; 3D view; checkCIF report

## Figures and Tables

**Fig. 1.**
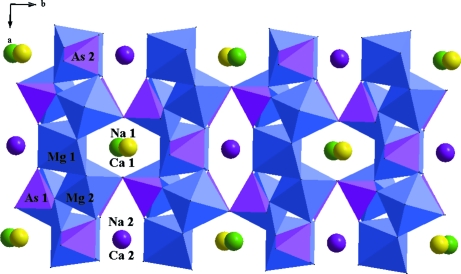
Projection of the structure of NaCaMg3(AsO4)3 along [001] direction.

**Fig. 2.**
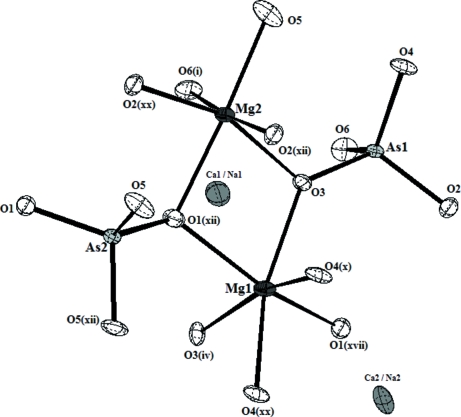
A view of a sheet showing the association mode of the MgO6 in chains with AsO4 tetrahedra shown with 50% probability displacement ellipsoids.

## supplementary crystallographic information

### Comment

The crystal structure of NaCaMg_3_(AsO_4_)_3_ is closely related to the common structure type of the well known mineral Alluaudite with the general formula *X*(1)*X*(2)*M*(1)*M*(2)_2_(PO_4_)_3_ (Moore, 1971; Yakubovitch *et al.*, 1994). It can be described by a Mg_3_(AsO_4_)_3_ framework built up by a complex arrangement of distorted MgO_6_ octahedra, and AsO_4_ tetrahedra. The projection of the structure in a polyhedral representation is presented in Fig. 1. It consists of Mg1O_6_ and Mg2O_6_ octahedra that share edges to form staggered chains stacked parallel to the [10–1] direction. Equivalent chains are linked together through the AsO_4_ tetrahedra corners. As1O_4_ connects two chains and thus two of its O atoms belong of the same chain. The As2O_4_ tetrahedron shares his four oxygen summits with four different MgO_6_ octahedra belonging to three adjacent chains, two belong to the same chain and the two others from two different chains. The arrangement of magnesium octahedra Mg1O_6_ and Mg2O_6_ in chains present a distortion, with the mean Mg1—O and Mg2—O distances 2.135Å and 2.079Å respectively. The O—Mg1—O angles range from 73.28° to 112.20°, whereas the O—Mg2—O angles vary between 77.62° to 108.84°. The two crystallographic distinct As atoms are surrounded by four O atoms with the mean distance of As1—O: 1.691Å and As2—O: 1.694 Å. This structural arrangement delimits two types of hexagonal tunnels, parallel to the *c* axis and located at (1/2, 0, *z*) and (0,0,*z*) respectively. Sodium Na1, Na2 and calcium Ca1, Ca2 cations are located in those channels. The structure is closely related to the alluaudite structure type. In NaCaMg_3_(AsO_4_)_3_, the *X*(1) site at (1/2, 0, 0) is empty. Whereas the *X*(2) site at (0, 0, 0) contains 0.30 Na2 and 0.70 Ca2. The site in the tunnel at (0, 0, *z*) shifted from the *X*(2) site by ± 0.25 along *z* is occupied by Na1 and Ca1 with respectively the occupation number 0.70 and 0.30. There are a number of possible models for the cationic distribution and it's not possible to decide which is the best solution. We retain the solution with same amount of sodium and calcium. First, the occupancies of Na1 and Ca1 in the site shifted from *X*(2) were refined. Second, for the site *X*(2), the occupancies of Na2 and Ca2 were fixed to obtain the electroneutrality. For each two cations in the same site the atomic displacement parameters were maintained the same with the instruction EADP. The bond valence sum of the Na1, Na2, Ca2, Mg1, Mg2, As1 and As2 atoms are in a good agreement with their oxidation states (Brown & Altermatt, 1985). For the calcium Ca1 which occupy partialy the tunnel the bond valence sums is different (1.33).

### Experimental

Single crystals of NaCaMg_3_(AsO_4_)_3_ were prepared by a mixture of NaNO_3_, CaCO_3_, MgN_2_O_6_(H_2_O)_6_ and NH_4_H_2_AsO_4_ with molar ratio of (1:1:2:3). The powder was ground, then heated in a porcelain crucible progressively until 1223 K. This temperature was held for 3 days. Then the mixture was cooled slowly to room temperature at 10 K/h. The product was washed with hot water. Prismatic and colorless crystals of the title compound were extracted. Their qualitative analysis by electron microscope probe revealed that it contains sodium, calcium, oxygen, arsenic and magnesium atoms.

### Crystal data

**Table:**

NaCaMg_3_(AsO_4_)_3_	*F*_000_ = 1048
*M**_r_* = 552.74	*D*_x_ = 3.870 Mg m^−^^3^
Monoclinic, *C*2/*c*	Mo *K*α radiation λ = 0.71073 Å
Hall symbol: -C 2yc	Cell parameters from 25 reflections
*a* = 11.880 (1) Å	θ = 2.4–28º
*b* = 12.817 (1) Å	µ = 11.36 mm^−^^1^
*c* = 6.741 (2) Å	*T* = 293 (2) K
β = 112.45 (1)º	Prismatic, colourless
*V* = 948.7 (3) Å^3^	0.6 × 0.2 × 0.15 mm
*Z* = 4	

### Data collection

**Table:**

Enraf–Nonius CAD-4 diffractometer	*R*_int_ = 0.021
Radiation source: fine-focus sealed tube	θ_max_ = 28.0º
Monochromator: graphite	θ_min_ = 2.4º
*T* = 293(2) K	*h* = −15→14
ω/2θ scans	*k* = −1→16
Absorption correction: ψ scan(North *et al.*, 1968)	*l* = 0→8
*T*_min_ = 0.110, *T*_max_ = 0.180	2 standard reflections
1358 measured reflections	every 120 min
1154 independent reflections	intensity decay: 0.4%
1133 reflections with *I* > 2σ(*I*)	

### Refinement

**Table:**

Refinement on *F*^2^	Secondary atom site location: difference Fourier map
Least-squares matrix: full	*w* = 1/[σ^2^(*F*_o_^2^) + (0.0244*P*)^2^ + 8.0874*P*] where *P* = (*F*_o_^2^ + 2*F*_c_^2^)/3
*R*[*F*^2^ > 2σ(*F*^2^)] = 0.022	(Δ/σ)_max_ < 0.001
*wR*(*F*^2^) = 0.060	Δρ_max_ = 0.99 e Å^−^^3^
*S* = 1.29	Δρ_min_ = −1.00 e Å^−^^3^
1154 reflections	Extinction correction: SHELXL97 (Sheldrick, 2008), Fc^*^=kFc[1+0.001xFc^2^λ^3^/sin(2θ)]^-1/4^
100 parameters	Extinction coefficient: 0.0071 (4)
Primary atom site location: structure-invariant direct methods	

### Special details

**Table:**

Geometry. All e.s.d.'s (except the e.s.d. in the dihedral angle between two l.s. planes) are estimated using the full covariance matrix. The cell e.s.d.'s are taken into account individually in the estimation of e.s.d.'s in distances, angles and torsion angles; correlations between e.s.d.'s in cell parameters are only used when they are defined by crystal symmetry. An approximate (isotropic) treatment of cell e.s.d.'s is used for estimating e.s.d.'s involving l.s. planes.
Refinement. Refinement of *F*^2^ against ALL reflections. The weighted *R*-factor *wR* and goodness of fit *S* are based on *F*^2^, conventional *R*-factors *R* are based on *F*, with *F* set to zero for negative *F*^2^. The threshold expression of *F*^2^ > σ(*F*^2^) is used only for calculating *R*-factors(gt) *etc*. and is not relevant to the choice of reflections for refinement. *R*-factors based on *F*^2^ are statistically about twice as large as those based on *F*, and *R*- factors based on ALL data will be even larger.

### Fractional atomic coordinates and isotropic or equivalent isotropic displacement parameters (Å^2^)

**Table:**

	*x*	*y*	*z*	*U*_iso_*/*U*_eq_	Occ. (<1)
As1	0.26904 (3)	0.38625 (2)	0.37969 (5)	0.00207 (13)	
As2	0.5000	0.21057 (3)	0.2500	0.00248 (14)	
Mg1	0.5000	0.23783 (13)	0.7500	0.0043 (3)	
Mg2	0.78584 (10)	0.15824 (9)	0.37643 (18)	0.0030 (2)	
Na1	0.5000	0.511 (3)	0.7500	0.019 (2)	0.70
Ca1	0.5000	0.523 (3)	0.7500	0.019 (2)	0.30
Ca2	0.5000	0.0000	0.0000	0.0132 (3)	0.70
Na2	0.5000	0.0000	0.0000	0.0132 (3)	0.30
O1	0.4635 (2)	0.28350 (19)	0.0247 (4)	0.0063 (5)	
O2	0.2837 (2)	0.31609 (19)	0.1792 (4)	0.0061 (5)	
O3	0.3415 (2)	0.32937 (19)	0.6218 (4)	0.0053 (5)	
O4	0.1175 (2)	0.39767 (19)	0.3197 (4)	0.0074 (5)	
O5	0.6086 (2)	0.1221 (2)	0.2626 (4)	0.0105 (5)	
O6	0.3381 (2)	0.50307 (19)	0.3963 (4)	0.0082 (5)	

### Atomic displacement parameters (Å^2^)

**Table:**

	*U*^11^	*U*^22^	*U*^33^	*U*^12^	*U*^13^	*U*^23^
As1	0.00076 (18)	0.00152 (19)	0.00403 (19)	0.00028 (10)	0.00101 (12)	0.00027 (10)
As2	0.0017 (2)	0.0013 (2)	0.0040 (2)	0.000	0.00054 (16)	0.000
Mg1	0.0020 (7)	0.0036 (7)	0.0080 (7)	0.000	0.0027 (6)	0.000
Mg2	0.0021 (5)	0.0022 (5)	0.0053 (5)	0.0000 (4)	0.0022 (4)	−0.0001 (4)
Na1	0.0091 (8)	0.035 (7)	0.0131 (8)	0.000	0.0032 (7)	0.000
Ca1	0.0091 (8)	0.035 (7)	0.0131 (8)	0.000	0.0032 (7)	0.000
Ca2	0.0161 (6)	0.0036 (5)	0.0107 (5)	−0.0026 (4)	−0.0051 (5)	−0.0003 (4)
Na2	0.0161 (6)	0.0036 (5)	0.0107 (5)	−0.0026 (4)	−0.0051 (5)	−0.0003 (4)
O1	0.0039 (11)	0.0094 (11)	0.0060 (11)	0.0020 (9)	0.0022 (9)	0.0025 (9)
O2	0.0080 (11)	0.0064 (11)	0.0045 (11)	0.0004 (9)	0.0031 (9)	−0.0010 (8)
O3	0.0050 (10)	0.0072 (11)	0.0033 (11)	0.0043 (9)	0.0010 (8)	0.0022 (8)
O4	0.0006 (10)	0.0065 (11)	0.0142 (12)	0.0005 (8)	0.0016 (9)	0.0000 (9)
O5	0.0034 (11)	0.0071 (11)	0.0169 (13)	0.0038 (9)	−0.0007 (9)	−0.0040 (9)
O6	0.0089 (11)	0.0022 (10)	0.0138 (12)	−0.0025 (9)	0.0047 (10)	−0.0007 (9)

### Geometric parameters (Å, °)

**Table:**

As1—O2	1.687 (2)	Na1—O1^ix^	2.99 (3)
As1—O6	1.690 (2)	Na1—O1^viii^	2.99 (3)
As1—O3	1.692 (2)	Ca1—O6	2.439 (5)
As1—O4	1.694 (2)	Ca1—O6^ii^	2.439 (5)
As2—O1^i^	1.693 (2)	Ca1—O6^viii^	2.497 (6)
As2—O1	1.693 (2)	Ca1—O6^ix^	2.497 (6)
As2—O5^i^	1.695 (2)	Ca1—O1^ix^	2.85 (4)
As2—O5	1.695 (2)	Ca1—O1^viii^	2.85 (4)
Mg1—O3^ii^	2.104 (2)	Ca1—O3	3.04 (4)
Mg1—O3	2.104 (2)	Ca1—O3^ii^	3.04 (4)
Mg1—O1^iii^	2.139 (2)	Ca2—O5	2.346 (3)
Mg1—O1^i^	2.139 (2)	Ca2—O5^x^	2.346 (3)
Mg1—O4^iv^	2.164 (3)	Ca2—O4^vii^	2.453 (3)
Mg1—O4^v^	2.164 (3)	Ca2—O4^xi^	2.453 (3)
Mg2—O5	2.001 (3)	Ca2—O4^xii^	2.538 (3)
Mg2—O3^vi^	2.068 (3)	Ca2—O4^vi^	2.538 (3)
Mg2—O6^vii^	2.072 (3)	Ca2—O5^i^	2.872 (3)
Mg2—O2^v^	2.077 (3)	Ca2—O5^xiii^	2.872 (3)
Mg2—O1^v^	2.098 (3)	Na2—O5	2.346 (3)
Mg2—O2^i^	2.163 (3)	Na2—O5^x^	2.346 (3)
Na1—O6	2.428 (3)	Na2—O4^vii^	2.453 (3)
Na1—O6^ii^	2.428 (3)	Na2—O4^xi^	2.453 (3)
Na1—O6^viii^	2.481 (4)	Na2—O4^xii^	2.538 (3)
Na1—O6^ix^	2.481 (4)	Na2—O4^vi^	2.538 (3)
Na1—O3	2.91 (3)	Na2—O5^i^	2.872 (3)
Na1—O3^ii^	2.91 (3)	Na2—O5^xiii^	2.872 (3)
			
O2—As1—O6	109.24 (12)	O6—Ca1—O6^ix^	92.2 (3)
O2—As1—O3	111.91 (11)	O6^ii^—Ca1—O6^ix^	86.1 (2)
O6—As1—O3	105.22 (12)	O6—Ca1—O1^ix^	121.1 (13)
O2—As1—O4	106.26 (12)	O6^ii^—Ca1—O1^ix^	70.7 (6)
O6—As1—O4	112.57 (12)	O6^viii^—Ca1—O1^ix^	83.7 (7)
O3—As1—O4	111.73 (12)	O6^ix^—Ca1—O1^ix^	110.2 (10)
O1^i^—As2—O1	112.96 (17)	O6—Ca1—O1^viii^	70.7 (6)
O1^i^—As2—O5^i^	110.10 (13)	O6^ii^—Ca1—O1^viii^	121.1 (13)
O1—As2—O5^i^	113.27 (12)	O6^viii^—Ca1—O1^viii^	110.2 (10)
O1^i^—As2—O5	113.27 (12)	O6^ix^—Ca1—O1^viii^	83.7 (7)
O1—As2—O5	110.10 (13)	O6^ii^—Ca1—O3	111.3 (13)
O5^i^—As2—O5	96.04 (18)	O6^viii^—Ca1—O3	60.9 (6)
O3^ii^—Mg1—O3	112.20 (15)	O6^ix^—Ca1—O3	105.3 (12)
O3^ii^—Mg1—O1^iii^	86.41 (10)	O6—Ca1—O3^ii^	111.3 (13)
O3—Mg1—O1^iii^	75.96 (9)	O6^viii^—Ca1—O3^ii^	105.3 (12)
O3^ii^—Mg1—O1^i^	75.96 (9)	O6^ix^—Ca1—O3^ii^	60.9 (6)
O3—Mg1—O1^i^	86.41 (10)	O3—Ca1—O3^ii^	70.2 (9)
O3—Mg1—O4^iv^	87.51 (9)	O5—Ca2—O4^vii^	74.30 (9)
O1^iii^—Mg1—O4^iv^	94.61 (10)	O5^x^—Ca2—O4^vii^	105.70 (9)
O1^i^—Mg1—O4^iv^	111.02 (10)	O5—Ca2—O4^xi^	105.70 (9)
O3^ii^—Mg1—O4^v^	87.51 (9)	O5^x^—Ca2—O4^xi^	74.30 (9)
O3—Mg1—O4^v^	159.81 (11)	O5—Ca2—O4^xii^	103.17 (9)
O1^iii^—Mg1—O4^v^	111.02 (10)	O5^x^—Ca2—O4^xii^	76.83 (9)
O1^i^—Mg1—O4^v^	94.61 (10)	O4^vii^—Ca2—O4^xii^	62.31 (10)
O4^iv^—Mg1—O4^v^	73.28 (14)	O4^xi^—Ca2—O4^xii^	117.69 (10)
O5—Mg2—O3^vi^	108.84 (12)	O5—Ca2—O4^vi^	76.83 (9)
O5—Mg2—O6^vii^	92.76 (11)	O5^x^—Ca2—O4^vi^	103.17 (9)
O3^vi^—Mg2—O6^vii^	86.82 (11)	O4^vii^—Ca2—O4^vi^	117.69 (10)
O5—Mg2—O2^v^	90.41 (11)	O4^xi^—Ca2—O4^vi^	62.31 (10)
O6^vii^—Mg2—O2^v^	101.79 (11)	O5—Ca2—O5^i^	56.66 (10)
O3^vi^—Mg2—O1^v^	77.62 (10)	O4^vii^—Ca2—O5^i^	91.59 (8)
O6^vii^—Mg2—O1^v^	95.10 (11)	O4^xi^—Ca2—O5^i^	88.41 (8)
O2^v^—Mg2—O1^v^	82.14 (10)	O4^xii^—Ca2—O5^i^	64.46 (7)
O5—Mg2—O2^i^	82.74 (10)	O4^vi^—Ca2—O5^i^	115.54 (7)
O3^vi^—Mg2—O2^i^	90.36 (10)	O5^x^—Ca2—O5^xiii^	56.66 (10)
O2^v^—Mg2—O2^i^	82.81 (10)	O4^vii^—Ca2—O5^xiii^	88.41 (8)
O1^v^—Mg2—O2^i^	89.86 (10)	O4^xi^—Ca2—O5^xiii^	91.59 (8)
O6—Na1—O6^viii^	86.76 (12)	O4^xii^—Ca2—O5^xiii^	115.54 (7)
O6^ii^—Na1—O6^viii^	92.89 (13)	O4^vi^—Ca2—O5^xiii^	64.46 (7)
O6—Na1—O6^ix^	92.89 (13)	O5—Na2—O4^vii^	74.30 (9)
O6^ii^—Na1—O6^ix^	86.76 (12)	O5^x^—Na2—O4^vii^	105.70 (9)
O6—Na1—O3	59.6 (5)	O5—Na2—O4^xi^	105.70 (9)
O6^ii^—Na1—O3	116.0 (11)	O5^x^—Na2—O4^xi^	74.30 (9)
O6^viii^—Na1—O3	63.1 (5)	O5—Na2—O4^xii^	103.17 (9)
O6^ix^—Na1—O3	109.6 (10)	O5^x^—Na2—O4^xii^	76.83 (9)
O6—Na1—O3^ii^	116.0 (11)	O4^vii^—Na2—O4^xii^	62.31 (10)
O6^viii^—Na1—O3^ii^	109.6 (10)	O4^xi^—Na2—O4^xii^	117.69 (10)
O6^ix^—Na1—O3^ii^	63.1 (5)	O5—Na2—O4^vi^	76.83 (9)
O3—Na1—O3^ii^	73.7 (8)	O5^x^—Na2—O4^vi^	103.17 (9)
O6—Na1—O1^ix^	116.3 (11)	O4^vii^—Na2—O4^vi^	117.69 (10)
O6^ii^—Na1—O1^ix^	68.4 (5)	O4^xi^—Na2—O4^vi^	62.31 (10)
O6^viii^—Na1—O1^ix^	81.2 (6)	O4^vii^—Na2—O5^i^	91.59 (8)
O6^ix^—Na1—O1^ix^	106.4 (9)	O4^xi^—Na2—O5^i^	88.41 (8)
O6—Na1—O1^viii^	68.4 (5)	O4^xii^—Na2—O5^i^	64.46 (7)
O6^ii^—Na1—O1^viii^	116.3 (11)	O4^vi^—Na2—O5^i^	115.54 (7)
O6^viii^—Na1—O1^viii^	106.4 (9)	O4^vii^—Na2—O5^xiii^	88.41 (8)
O6^ix^—Na1—O1^viii^	81.2 (6)	O4^xi^—Na2—O5^xiii^	91.59 (8)
O6—Ca1—O6^viii^	86.1 (2)	O4^xii^—Na2—O5^xiii^	115.54 (7)
O6^ii^—Ca1—O6^viii^	92.2 (3)	O4^vi^—Na2—O5^xiii^	64.46 (7)

Symmetry codes: (i) −*x*+1, *y*, −*z*+1/2; (ii) −*x*+1, *y*, −*z*+3/2; (iii) *x*, *y*, *z*+1; (iv) −*x*+1/2, −*y*+1/2, −*z*+1; (v) *x*+1/2, −*y*+1/2, *z*+1/2; (vi) *x*+1/2, −*y*+1/2, *z*−1/2; (vii) *x*+1/2, *y*−1/2, *z*; (viii) *x*, −*y*+1, *z*+1/2; (ix) −*x*+1, −*y*+1, −*z*+1; (x) −*x*+1, −*y*, −*z*; (xi) −*x*+1/2, −*y*+1/2, −*z*; (xii) −*x*+1/2, *y*−1/2, −*z*+1/2; (xiii) *x*, −*y*, *z*−1/2.
